# Prospective Multicenter Study of Chemotherapy-Induced *Clostridium* (*Clostridioides) difficile* Infection in Patients With Lung Cancer: North Japan Lung Cancer Study Group Trial 1204

**DOI:** 10.3389/fonc.2021.685320

**Published:** 2021-07-15

**Authors:** Yukihiro Toi, Takao Kobayashi, Toshiyuki Harada, Taku Nakagawa, Yoshiaki Mori, Tomoya Kuda, Shunichi Sugawara

**Affiliations:** ^1^ Department of Pulmonary Medicine, Sendai Kousei Hospital, Sendai, Japan; ^2^ Department of Respiratory Medicine, Tohoku Medical and Pharmaceutical University, Sendai, Japan; ^3^ Department of Respiratory Medicine, Japan Community Health Care Organization Hokkaido Hospital, Sapporo, Japan; ^4^ Department of Thoracic Surgery, Omagari-Kosei Medical Center, Daisen, Japan; ^5^ Department of Pulmonary Medicine, Iwate Prefectural Central Hospital, Morioka, Japan; ^6^ Department of Pulmonary Medicine, Naha City Hospital, Naha, Japan

**Keywords:** *Clostridium* (*Clostridioides) difficile*, diarrhea, lung cancer, chemotherapy, prospective multicenter study

## Abstract

**Background:**

Diarrhea post-antibiotic use is primarily attributed to mucosal lesions induced by *Clostridium* (*Clostridioides) difficile* (*C. difficile*) infection (CDI). Cancer patients undergoing chemotherapy might have a higher risk of CDI even when prior antibiotics are not used. Thus far, the relationship between lung cancer chemotherapy and the incidence of diarrhea remains unclear. This prospective multicenter study aimed to determine the incidence of CDI in lung cancer patients undergoing chemotherapy.

**Methods:**

The presence of *C. difficile* and its toxins was investigated in lung cancer patients experiencing diarrhea during chemotherapy including paclitaxel (PTX), nanoparticle albumin-bound paclitaxel (nab-PTX), docetaxel (DOC), tegafur-gimeracil-oteracil (S-1), or irinotecan (CPT-11). If grade 2 or higher diarrhea occurred, then a stool culture was performed to detect anaerobic organisms and *C. difficile* toxins A and B. Additional data were collected through patient interviews and medical chart review.

**Results:**

A total of 263 consecutive patients were enrolled in the study; grade 2 or higher diarrhea was observed in 22 patients (8.4%); CDI was confirmed in five of them (1.9%). The incidence of CDI was 22.7% of all diarrhea cases, and 50% of patients treated with PTX were CDI positive; the incidence of CDI was significantly higher in patients treated with PTX (P=0.039). Among the diarrhea cases, CDI patients had significantly worse ECOG performance status (PS) (P=0.043) and a significantly higher neutrophil count (P=0.028) than non-CDI patients. No CDI patients received antibiotics before cancer chemotherapy.

**Conclusions:**

Although diarrhea does not always affect a large portion of lung cancer chemotherapy recipients, clinicians should consider the possibility of CDI occurrence in lung cancer patients receiving chemotherapy, particularly PTX, without prior antibiotic exposure.

## Introduction

*Clostridium* (*Clostridioides) difficile* (*C. difficile*) infection (CDI) is a common nosocomial infection associated with prior antibiotic use (1). Some patients remain asymptomatic after exposure to *C. difficile*, whereas others develop a range of illnesses, from mild diarrhea to fulminant colitis (2). Transmission of CDI occurs from one patient to the others and causes mild diarrhea. The risk of CDI can also be high when antibiotics disrupt host defenses provided by indigenous microflora in the colon. Potential risk factors for CDI include old age, underlying illness, poor infection control practice, prolonged hospitalization, and anticancer drugs (1). Despite great efforts made over the past ten years to manage the CDI burden (3–5), there are still gray areas in CDI management (1, 6). CDI accounted for almost half a million of the total infections and was associated with approximately 29,000 deaths in 2011 in the United States (6). In Japan, all-cause mortality in patients with CDI was reported to increase from 3.4% to 15.1% between 2007 and 2013 (7).

Lung cancer is the leading cause of cancer-related mortality worldwide (8). With an increase in the number of hospital admissions related to either the underlying malignancy or comorbidities (9), the frequencies of prolonged admission, continuous antibiotic use, and general patient weakness have also increased. Chemotherapeutic agents can directly damage the intestinal mucosa (enterocolitis) and cause changes in the intestinal microflora, leading to mucosal damage diarrhea. Occasionally, damage caused by neutropenia from their use contributes to the development of CDI (10–12). Therefore, lung cancer patients could be at a high risk of CDI. However, only few studies to date have investigated the relationship between lung cancer chemotherapy and the incidence of diarrhea. A retrospective study reported that CDI was diagnosed in 44 of 188 (23.4%) lung cancer patients (13). Our previous single-institutional retrospective study reported that tegafur-gimeracil-oteracil (S-1) and irinotecan (CPT-11) tended to increase the rate of CDI (14). Several studies have investigated the incidence of CDI in patients treated with paclitaxel (15–17). Severe CDI cases associated with docetaxel have also been reported (18).

This prospective multicenter study aimed to determine the incidence of CDI among lung cancer patients receiving chemotherapy.

## Patients and Methods

Patients older than 20 years with histologically or cytologically proven lung cancer treated with anticancer drugs, including paclitaxel (PTX), nanoparticle albumin-bound paclitaxel (nab-PTX), docetaxel (DOC), tegafur-gimeracil-oteracil (S-1), or irinotecan (CPT-11), were eligible for this study. Other eligibility criteria included Eastern Cooperative Oncology Group performance status 0–3 and an estimated life expectancy of ≥3 months. Patients with psychotic disease, ileus, CDI onset within two months, or diarrhea were excluded. If we noted grade 2 or higher diarrhea, as defined using the Common Terminology Criteria for Adverse Events Version 4 (CTC), stool culture was performed to detect anaerobic organisms and toxins A and B. A diagnosis of CDI was made on the basis of a combination of clinical and laboratory findings (19). The following criteria are commonly used in CDI diagnosis: (1) the presence of diarrhea (defined as the passage of three or more unformed stools in 24 or fewer consecutive hours); and (2) a stool test positive for the presence of toxigenic *C. difficile* or its toxins or colonoscopic or histopathologic findings demonstrating pseudomembranous colitis (19). A colonoscopic diagnosis of pseudomembranous enteritis is not necessarily required for CDI diagnosis. CDI treatment was not limited; diarrhea prophylaxis or laxatives use was not prohibited. After diarrhea resolved, patient assessment was terminated. If no diarrhea is observed four weeks after completing scheduled chemotherapy, patient assessment was terminated. Further data were collected through patient interviews and medical chart review.

This study was approved by the Institutional Review Board (IRB) and Ethical Review Committee (ERC) at each institution and conducted in compliance with Good Clinical Practice (GCP) and Declaration of Helsinki. This trial was registered with the University Hospital Medical Information Network (UMIN), number UMIN C000008432. Written informed consent was obtained from all enrolled patients.

### Statistical Analysis

Categorical variables were tested for significance using Fisher’s test, Student’s t test, the Mann-Whitney U test, or Welch’s t test, as appropriate. All p values were two-sided, with a value of <.05 considered to indicate statistical significance. All statistical analyses were performed using EZR (Saitama Medical Centre, Jichi Medical University, Saitama, Japan), a graphical user interface for R (The R Foundation for Statistical Computing, Vienna, Austria). EZR is a modified version of R commander and is designed to add statistical functions frequently used in biostatistics (20).

## Results

**Figure 1:**
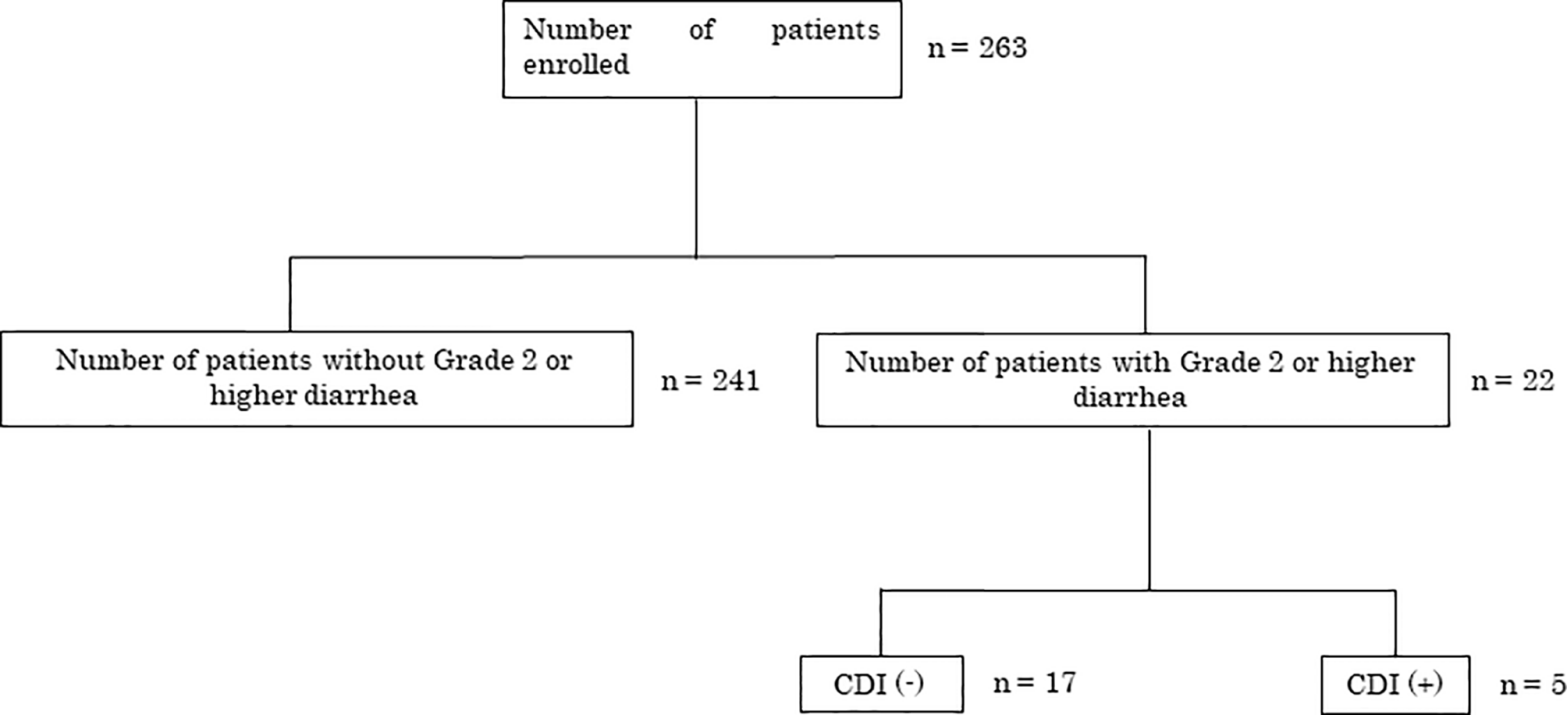
Patient enrollment and outcomes. CDI, *Clostridium* (*Clostridioides*) *difficile* infection.

From October 2012 to August 2014, 263 consecutive patients were enrolled from six institutions in Japan. Diarrhea of grade 2 or higher was observed in 22/263 (8.4%) patients. *C. difficile* toxins A or B were present in 5/22 patients, indicating CDI diagnosis (**Figure 1**).

**Table 1** shows the clinical characteristics of patients in this study. Among 22 patients who developed diarrhea, patients with CDI had significantly worse ECOG performance status (PS) and a significantly higher neutrophil count than those without CDI (P=0.043 and P=0.028). No association was found between age, sex, prior chemotherapy administration, antibiotic administration within one month, minimum lymphocyte count, serum albumin level, medication (proton pump inhibitor, laxative, histamine 2 blocker, intestinal drugs), and diarrhea. CDI in all patients was mild, and treatment with oral metronidazole immediately resolved. There was no evidence of nosocomial transmission of *C. difficile*.

**Table 1:** Patient characteristics and results.

	All patients (n=263)	Without diarrhea (n=241)	With diarrhea (n=22)		
			Without CDI (n=17)	With CDI (n=5)	P
**Age (median)**	43-88 (67)	46-86 (67)	43-78 (64)	59-78 (68)	0.658^a^
**Male/female**	202/61	188/53	11/6	3/2	1.00^b^
**ECOG (PS)** **(0/1/2/3)**	151/93/15/4	139/85/14/3	11/6/0/0	1/2/1/1	0.043^b^
**Prior chemotherapy (yes/no)**	154/109	141/100	11/6	1/4	0.135^b^
**Admission within one year (yes/no)**	151/112	138/103	11/6	2/3	0.609^b^
**Antibiotic administration within one month (yes/no)**	12/251	10/231	3/14	0/5	1.00^b^
**Minimum neutrophil count: median (/mm^3^)**	21-11230 (940)	21-11230 (968)	207-1760 (722)	250-2325 (1360)	0.028^c^
**Minimum lymphocyte count: median (/mm^3^)**	12-2330 (730)	12-2330 (750)	51-1350 (600)	13-880 (390)	0.249^d^
**Serum** **albumin: median (g/dl)**	1.3-4.3 (2.9)	1.3-4.3 (3.0)	1.8-3.8 (2.7)	1.9-2.6 (2.3)	0.061^c^
**Proton pump inhibitor or histamine 2 blocker**	100	79	9	2	1.00^b^
**Laxative**	53	49	4	0	1.00^b^
**Intestinal drugs**	25	12	3	0	1.00^b^

CDI, Clostridium (Clostridioides) difficile infection; ECOG PS, Eastern Cooperative Oncology Group performance status.

a Results calculated with Student’s t test.

b Results calculated with Fisher’s exact test.

c Results calculated with Student’s t test.

d Results calculated with Mann-Whitney U test.

**Table 2** shows the relationship between each drug and the incidence of CDI. Each anticancer drug was used either alone or in combination with another one. At the time of CDI diagnosis, no patients had prior antibiotics within one month. Twenty-two patients had grade 2 or higher diarrhea. Diarrhea of grade 2 or higher was observed in 8/102 (7.8%) patients treated with PTX, 5/58 (8.6%) patients treated with DOC, 4/62 (6.4%) patients treated with S-1, and 9/51 (17.6%) patients treated with CPT-11. No diarrhea developed in patients treated with nab-PTX. The incidence of CDI was significantly higher in patients who received PTX (P=0.039). There was no association between the use of other drugs and the development of CDI.

**Table 2:** Relationship between each drug and CDI.

Chemotherapy agent (alone or combination)	All patients (n=263)	With diarrhea (n=22)		
		Without CDI (n=17)	With CDI (n=5)	p
PTX	102	4	4	0.039^a^
Nab-PTX	8	0	0	NA
DOC	58	5	0	0.29^a^
S-1	62	4	0	0.54^a^
CPT-11	51	8	1	0.36^a^

CDI, Clostridium (Clostridioides) difficile infection, PTX, paclitaxel; nab-PTX, nanoparticle albumin-bound paclitaxel; DOC, docetaxel; S-1, tegafur-gimeracil-oteracil; CPT-11, irinotecan.

Each anticancer drug was used alone or in combination with another one.

a Results calculated with Fisher’s exact test.

**Table 3** shows the diarrhea onset time. Overall, the median number of days until the onset of grade 2 or higher diarrhea was 7 days (range 0–67). However, in patients without CDI, the median number of days until onset was 7 days (range 0–29), and the median number of days until onset was 38 days (range, 10–67) in patients with CDI. Patients who developed CDI tended to have a later onset date (P=0.084).

**Table 3:** Diarrhea onset time.

	Median duration (days)	
**All diarrhea cases**	7 (0-67)	
**CDI (+)**	38 (10-67)	P=0.084^a^
**CDI (−)**	7 (0-29)	

CDI, Clostridium (Clostridioides) difficile infection; CDI (+), patients with lung cancer with CDI; CDI (-), patients with lung cancer without CDI.

a Results calculated with Welch’s t test.

## Discussion

This study, to the best of our knowledge, is the first prospective multicenter study to assess the relationship of lung cancer chemotherapy with diarrhea and CDI. We found that the incidence of CDI was 22.7% of all diarrhea cases in patients with lung cancer treated with anticancer drug.

Rodríguez Garzotto et al. reported that they could not find any association between a particular type of chemotherapy and CDI in oncology patients (10). However, in the present study, the incidence of CDI among diarrhea cases was significantly higher in patients who received PTX (P=0.039).

Hwang et al. reported that albumin levels were significantly lower, and PS score was significantly higher in lung cancer patients with CDI than in those without CDI (13). In the present study, there was a similar trend of poor PS and low albumin levels in CDI-positive diarrhea cases.

In the present study, patients who developed CDI tended to have a later onset date. In one report, the median interval from completing a treatment course to CDI diagnosis was 20.3 days (21). The onset of CDI symptoms may occur immediately after chemotherapeutic agents’ initiation or may be delayed.

The exact etiology of chemotherapy-induced CDI is unclear. *C. difficile* is a spore-forming anaerobe that can survive for several months in air as spores. It is resistant to gastric acid and pathogenic when it reaches the intestinal tract. There has a high carrier rate among hospital inpatients (22). *C. difficile* is the most common cause of nosocomial diarrhea worldwide. Several potential risk factors for CDI have been reported, but the use of antibiotics is particularly　representative. In this study, none of the five CDI patients had received antibiotics within a month, and no nosocomial infections were identified. We believe that the administration of anticancer drugs may affect the development of CDI.

The American Society of Clinical Oncology (ASCO) guidelines on the treatment of chemotherapy-induced diarrhea recommend starting treatment with an anti-diarrheal agent or antibiotic together with testing *C. difficile* as part of a stool work-up if symptoms do not improve (23). In the case of antibiotic-associated diarrhea, early screening for *C. difficile* should be performed aggressively using stool culture to account for the possibility of CDI. Therefore, in the case of post-chemotherapy diarrhea, as with antibiotic-associated diarrhea, we believe that it is important to perform stool testing for *C. difficile* at an earlier stage. Further, close cooperation between clinicians who administer chemotherapy and hospital infection control teams is important for cancer treatment. As for treatment, metronidazole, vancomycin, and fidaxomicin are available. Recurrence has been reported in up to 25% of people. Tentatively, it has been shown that fecal microbiota transplantation and probiotics may reduce the risk of recurrence.

Recently, immune-checkpoint inhibitors have become an important part of treatment for lung cancer, and both single-agent immune checkpoint inhibitors and combination therapy with immune checkpoint inhibitors and anticancer drugs are standard first-line treatments (24, 25). The therapy causes various adverse effects, including colitis. It is reported to range from 9% to 12% (26–28). In particular, several combination therapies of immune checkpoint inhibitors and anticancer drugs including PTX have been approved, and we believe that the results of this study are important for future lung cancer treatment (29–31).

The major limitation of the study is the small number of CDI cases.

The results of this study showed that approximately a quarter of the diarrhea cases were positive for CDI.　When patients show grade 2 or higher diarrhea after treatment with anticancer agents, especially with PTX, the possibility of CDI must be considered. Testing for *C. difficile* should be carried out at an early stage, even without prior antibiotic exposure.

## Data Availability Statement

The original contributions presented in the study are included in the article/supplementary files, further inquiries can be directed to the corresponding author at yuktoi119@yahoo.co.jp.

## Ethics Statement

This study was approved by the Institutional Review Board (IRB) and Ethical Review Committee (ERC) at each institution and conducted in compliance with Good Clinical Practice (GCP) and Declaration of Helsinki. This trial was registered with the University Hospital Medical Information Network (UMIN), number UMIN C000008432. Written informed consent was obtained from all enrolled patients. The patients/participants provided their written informed consent to participate in this study.

## Author Contributions

YT and TKo has full access to all of the data in the study and takes responsibility for the integrity of the data and the accuracy of data analysis.　Study concept and design: TKo and SS. Acquisition, analysis, or interpretation of data: TKo, YT, TH, TN, YM, TKu, and SS. Drafting of the manuscript: TKo, YT, and SS. Critical revision of the manuscript for important intellectual content: TKo, YT, TH, TN, YM, TKu, and SS. Statistical analysis: TKo and YT. Study supervision: SS. All authors contributed to the article and approved the submitted version.

## Conflict of Interest

SS reports lecture fees from Ono Pharmaceutical, Bristol-Myers Squibb, MSD, AstraZeneca, Chugai Pharma, Nippon Boehringer Ingelheim, Pfizer, Taiho Pharmaceutical, Eli Lilly and Company, Novartis, Yakult Honsha, and Kyowa Hakko Kirin. YT reports lecture fees from Ono Pharmaceutical, Bristol-Myers Squibb, MSD, AstraZeneca, and Taiho Pharmaceutical.　

The remaining authors declare that the research was conducted in the absence of any commercial or financial relationships that could be construed as a potential conflict of interest.
